# Impact of Mid-to-Late Gestational Overfeeding on Maternal Performance and Calf Outcomes in Hanwoo Cattle: A Machine Learning Approach

**DOI:** 10.3390/ani16121902

**Published:** 2026-06-19

**Authors:** Myungsun Park, Borhan Shokrollahi, Gi Suk Jang, Shil Jin, Sung Jin Moon, Kyung Hwan Um, Sun Sik Jang, Youl Chang Baek

**Affiliations:** 1 Hanwoo Research Center, National Institute of Animal Science (NIAS), Rural Development Administration (RDA), Pyeongchang 25340, Republic of Korea; sunnypark411@korea.kr (M.P.); shinnanda16@naver.com (G.S.J.); jins21@korea.kr (S.J.); moonsj27@korea.kr (S.J.M.); umkh9969@korea.kr (K.H.U.); 2 Subtropical Livestock Research Center, National Institute of Animal Science, Rural Development Administration (RDA), Jeju 63242, Republic of Korea; borhansh@gmail.com

**Keywords:** Hanwoo cattle, gestational overfeeding, calf performance, metabolic parameters, machine learning

## Abstract

This study evaluated the effects of maternal overfeeding during mid-to-late gestation on maternal productivity, metabolic status, reproductive recovery, and calf performance in Hanwoo cattle using conventional statistics and machine learning (ML) approaches. A total of 243 pregnant cows were assigned to either a control group or an overfeeding group from gestation day 90 to parturition. The overfeeding treatment increased nutrient supply to approximately 140–145% of the control level. Maternal body weight (BW), body condition score (BCS), serum metabolites, and reproductive traits were evaluated throughout gestation and postpartum, while calf growth, morphometrics, and metabolic traits were assessed at birth and weaning. Calves were further classified into growth- or meat-quality-oriented genotypes using SNP-based profiling. Overfeeding increased maternal BW gain and BCS during gestation and reduced circulating non-esterified fatty acid concentrations, indicating improved maternal energy status. However, overfed cows showed a longer interval to postpartum estrus return. Calf birth weight was not significantly affected by maternal overfeeding, whereas calf growth and morphometric traits at weaning were more strongly influenced by parity, sex, and genotype. Machine learning models identified gestational BW, metabolic indicators, calf feed intake, and genotype as major predictors of maternal and calf outcomes, with random forest and XGBoost showing superior predictive performance compared with linear models. These findings suggest that parity- and genotype-informed nutritional management combined with ML-based prediction may support precision feeding strategies in beef cattle production systems.

## Simple Summary

Maternal nutrition during gestation can influence metabolic adaptation, reproductive recovery, and calf development in beef cattle. In this study, overfeeding during mid-to-late gestation in Hanwoo cattle improved maternal body condition and altered metabolic responses, whereas calf growth performance was influenced more strongly by parity, sex, and genotype than by maternal nutritional treatment alone. Machine learning models identified gestational body weight, metabolic indicators, feed efficiency, and calf genotype as important predictors of maternal and calf productivity-related traits. These findings suggest that parity- and genotype-informed nutritional management combined with machine learning approaches may support precision feeding strategies in beef cattle production systems.

## 1. Introduction

Maternal nutrition during pregnancy plays a critical role in determining both maternal physiology and offspring performance in beef cattle [1,2]. In particular, nutritional imbalance during mid-to-late gestation can influence maternal energy metabolism, postpartum reproductive recovery, and calf growth and development [3,4,5]. Hanwoo cattle (Bos taurus coreanae) are a native Korean beef breed well known for superior marbling ability, meat quality, and strong consumer preference in East Asian beef markets. In many beef production systems, cows are exposed to seasonal fluctuations in forage quality during gestation, which may result in insufficient nutrient intake and impaired maternal and fetal performance [6,7,8]. Under such conditions, dietary total digestible nutrients (TDN) and crude protein levels may fail to meet the increasing metabolic demands associated with fetal growth and maternal maintenance [9,10]. Previous studies have shown that strategic nutritional supplementation during gestation can improve dry matter intake (DMI), nutrient utilization, body weight (BW), and body condition score (BCS) in beef cows [8,11].

Late gestation is particularly important because approximately 70–75% of fetal growth occurs during the final trimester of pregnancy [12,13,14]. During this period, key developmental processes such as skeletal muscle formation, adipogenesis, and immune system maturation are highly sensitive to maternal nutrient supply [13,14,15]. Consequently, maternal nutritional status during gestation has been associated with long-term effects on offspring growth, metabolism, and productive performance through fetal programming mechanisms [2,14,16].

Despite increasing interest in gestational nutrition, previous studies have reported inconsistent responses in calf growth and reproductive performance following maternal supplementation [17,18,19]. Some studies demonstrated improvements in offspring growth and metabolic status, whereas others reported limited or negligible effects on birth weight or postnatal performance [18,19,20]. These discrepancies suggest that maternal nutrition alone may not fully explain variation in offspring outcomes. Biological factors such as parity, maternal age, metabolic status, and calf genotype may also substantially contribute to developmental and productive responses [21,22,23].

Previous studies have applied machine learning approaches to predict body weight, feed efficiency, reproductive traits, and metabolic responses in livestock production systems, demonstrating their utility for analyzing complex biological datasets [24,25,26,27,28]. Conventional statistical approaches are often limited in their ability to capture complex nonlinear relationships among nutritional, physiological, and genetic variables [24]. In contrast, machine learning (ML) approaches have recently demonstrated strong predictive performance for livestock growth, metabolic, and reproductive traits [25,26,27,28], enabling the identification of influential predictors and latent relationships within large biological datasets and providing new opportunities for precision livestock management.

Therefore, the objectives of this study were to evaluate the effects of mid-to-late gestational overfeeding on maternal BW, BCS, reproductive recovery, metabolic indicators, and calf growth performance in Hanwoo cattle, and to identify important predictors associated with these outcomes using machine learning approaches. We hypothesized that maternal overfeeding during gestation would influence postpartum maternal condition and calf development, while parity, metabolic status, and calf genotype would also contribute substantially to variation in productivity-related traits.

## 2. Materials and Methods

### 2.1. Animals, Housing, and Experimental Design

This study was conducted at the Hanwoo Research Center, National Institute of Animal Science (NIAS), Republic of Korea. A total of 243 pregnant Hanwoo cows (Bos taurus coreanae) were enrolled in the experiment. Prior to artificial insemination (AI), all animals were maintained under standardized feeding and management conditions according to the Korean Feeding Standard for Hanwoo [10].

Estrous cycles were synchronized, and AI was performed using commercially available semen. Pregnancy was confirmed at 90 d after insemination using transrectal palpation and pregnancy diagnostic kits. After pregnancy confirmation, cows were randomly assigned to either the control (CON; *n* = 123) or overfeeding (OVF; *n* = 120) treatment group. Dietary treatments were applied from gestation day 90 until parturition. A total of 243 pregnant Hanwoo cows with complete reproductive and experimental records were included in the final analyses. Animals with pregnancy loss, abortion, or incomplete datasets were excluded prior to statistical analysis.

Animals were housed in individual pens with free access to fresh water and mineral blocks throughout the experimental period.

### 2.2. Nutritional Treatments and Diet Composition

The CON group received 3.0 kg concentrate and 5.0 kg rice straw daily, which met the maintenance nutrient requirements of a 400 kg Hanwoo cow. The OVF group received 4.5 kg concentrate and 6.5 kg rice straw daily, corresponding to approximately 140–145% of the nutrient supply provided to the CON group on a dry matter intake (DMI), total digestible nutrient (TDN), and crude protein (CP) basis.

The OVF treatment increased concentrate allowance to 150% of the control level on an as-fed basis. However, actual nutrient intake corresponded to approximately 140–145% of the control group because intake calculations were based on measured feed refusals and dry matter intake. The dietary level was selected based on previous studies demonstrating that increased maternal energy and nutrient supply during gestation may promote fetal adipogenic development and metabolic programming in beef cattle offspring [12,13], while also reflecting practical overfeeding conditions under commercial Hanwoo production systems. Estimated nutrient intake was 6.07 vs. 8.42 kg/d for DMI, 4.07 vs. 5.69 kg/d for TDN, and 0.65 vs. 0.93 kg/d for CP in the CON and OVF groups, respectively.

Feed ingredients and chemical composition were analyzed according to AOAC procedures [29]. Nutrient composition of the concentrate and rice straw is presented in Table 1.

### 2.3. Feeding and Management Protocols

Feed was offered twice daily, and refusals were recorded daily for each individual animal. DMI was calculated based on the dry matter composition of the diets and orts. Body weight (BW) and body condition score (BCS) were monitored throughout gestation and postpartum.

Calves remained with their dams until weaning at 90 d of age. From two weeks after birth, calves were provided ad libitum access to a commercial starter diet and high-quality roughage formulated according to NIAS recommendations [10]. Fresh water was freely available throughout the rearing period. This management strategy was applied to promote early rumen development and facilitate adaptation to post-weaning feeding conditions.

### 2.4. Genotyping and Genotype Classification of Calves

To classify calves according to genetic potential, SNP-based genotyping was conducted using markers associated with growth and marbling traits in Hanwoo cattle [30,31]. Genomic DNA was extracted from peripheral blood samples using a commercial genomic DNA extraction kit (Qiagen, Hilden, Germany) according to the manufacturer’s instructions.

Based on genomic breeding values, calves were categorized into either a growth-oriented genotype group or a meat-quality-oriented (MQ) genotype group. Genotype classification was subsequently included as a covariate in regression and machine learning analyses.

### 2.5. Data Collection and Laboratory Analyses

#### 2.5.1. Dam Measurements

Dam BW and BCS were recorded at AI, gestation day 60, day 180, day 270, and 2 months postpartum. Body condition score was evaluated using a standardized 5-point scoring system by trained personnel.

Blood samples were collected from the jugular vein at gestation days 90, 180, and 270, and at 1 d postpartum. Samples were centrifuged at 2000× *g* for 20 min, and serum was stored at −80 °C until analysis.

Serum metabolites, including glucose (Glu), cholesterol (Cho), triglycerides (TG), blood urea nitrogen (BUN), total protein (TP), albumin (Alb), and non-esterified fatty acids (NEFA), were analyzed using an automated biochemical analyzer (Hitachi 7180, Tokyo, Japan). Serum estradiol-17β concentrations were determined using a commercial bovine ELISA kit (Estradiol (Bovine) ELISA Kit, Cat. No. KA2276, Abnova Corporation, Taipei, Taiwan) according to the manufacturer’s instructions. The assay was based on a competitive enzyme immunoassay, and absorbance was measured at 450 nm. The analytical detection limit of the assay was 5 pg/mL.

#### 2.5.2. Calf Measurements

Calf BW, average daily gain (ADG), and morphometric traits were evaluated at birth and weaning (90 d). Morphometric measurements included withers height, body length, chest girth, chest depth, pelvic width, hip width, rump width, and rump length. Morphometric traits were measured using a calibrated livestock measuring stick and flexible measuring tape according to standardized cattle measurement procedures.

Blood samples were collected from calves at birth and weaning using heparinized vacutainer tubes. Serum metabolite analyses were performed using the same procedures described for dams.

### 2.6. Statistical Analysis

#### Data Preprocessing and Descriptive Statistics

All statistical analyses were performed in R software (version 4.3.3; R Foundation for Statistical Computing, Vienna, Austria) [32].

Prior to analysis, data distributions were assessed using the Shapiro–Wilk test and visual inspection of residual plots. Variables that deviated from normality were log-transformed when necessary.

Parity was classified into four categories: heifer (0), young (1–2 parity), mature (3–5 parity), and older (≥6 parity). Treatment group, dam age, calf sex, and calf genotype were included as explanatory variables.

To evaluate treatment effects on maternal and calf traits, robust linear regression models were fitted using the rlm() function of the MASS package in R to reduce sensitivity to heteroscedasticity and outliers [33].

The statistical model for maternal traits was:

$$Y_{ij}=\;\mu +{Treatment}_{i}+{Parity}_{i}+{DamAge}_{i}+\varepsilon_{ij}$$

For calf traits, the following model was used:

$$Y_{ijk}=\mu +{Treatment}_{i}+{Parity}_{i}{\;+\;Sex}_{i}+{Genotype}_{k}+{DamAge}_{i}+\varepsilon_{ijk}$$

Statistical significance was defined as *p* < 0.05.

### 2.7. Machine Learning Analysis

Machine learning analyses were conducted to evaluate predictive performance and identify influential predictors associated with maternal and calf outcomes.

Datasets were randomly divided into training (75%) and testing (25%) subsets using a fixed random seed. The 75%/25% training–testing split was selected because it is commonly used to balance model training efficiency and independent performance evaluation in biological datasets. Random forest and XGBoost were selected because they are widely used ensemble-based machine learning algorithms capable of capturing nonlinear interactions and complex biological relationships in livestock datasets while maintaining relatively high interpretability. Hyperparameter settings for random forest and XGBoost were selected based on preliminary tuning procedures and commonly applied configurations reported in previous livestock prediction studies. Three predictive models were evaluated: linear regression (LM), random forest (RF), and extreme gradient boosting (XGBoost).

Random forest models were fitted using 500 trees with mtry = 3. XGBoost models were trained using a maximum tree depth of 4, learning rate of 0.1, and subsampling ratio of 0.8. Early stopping was applied when test-set performance did not improve for 10 consecutive iterations.

Model performance was evaluated using root mean squared error (RMSE) and coefficient of determination (R^2^). Variable importance was estimated using %IncMSE for RF and gain values for XGBoost.

To minimize information leakage, only biologically relevant variables available prior to or contemporaneous with each target outcome were included during model development. Postnatal growth-related variables were included only for prediction of weaning-associated outcomes.

## 3. Results

### 3.1. Data Preprocessing and Outcome Transformation

Data from 243 cow–calf pairs were included in the analyses. Several outcome variables showed non-normal distributions based on the Shapiro–Wilk test and residual diagnostics. Variables with skewed distributions were log-transformed before analysis. Missing numeric values were imputed according to the proportion of missingness as described in the Materials and Methods section. After preprocessing, model diagnostics indicated improved variance stability and residual distribution.

### 3.2. Maternal Performance and Metabolic Responses

#### 3.2.1. Body Weight, Body Condition Score, and Growth Performance

Maternal parity and nutritional treatment significantly influenced body weight (BW), body condition score (BCS), and growth performance during gestation (Table 2). Younger-parity cows showed greater BW during mid and late gestation and after parturition compared with older cows (*p* < 0.05). Overfeeding increased BW during late gestation (*p* < 0.001).

Overfed cows exhibited higher BCS during both mid and late gestation than control cows (*p* < 0.001). Average daily gain (ADG) from mid-to-late gestation was greater in the overfeeding group (*p* < 0.01), whereas increasing dam age was associated with reduced ADG (*p* < 0.001). Feed conversion ratio (FCR) during gestation was lower in overfed cows and in younger-parity cows, indicating greater feed efficiency (*p* < 0.01).

The interval to postpartum estrus return was longer in overfed cows than in control cows (*p* < 0.01).

#### 3.2.2. Metabolic and Hormonal Parameters

Several metabolic indicators were affected by nutritional treatment, parity, and dam age (Table 2). Glucose concentrations during late gestation were lower in overfed cows than in controls (*p* < 0.001). Cholesterol concentrations during mid and late gestation were higher in the overfeeding group (*p* < 0.05).

Non-esterified fatty acid (NEFA) concentrations during mid and late gestation were lower in overfed cows (*p* < 0.05), suggesting reduced lipid mobilization. Blood urea nitrogen (BUN) and triglyceride concentrations were positively associated with dam age (*p* < 0.05). Total protein concentrations during late gestation were greater in mature-parity cows (*p* < 0.05).

Progesterone concentrations during mid gestation were lower in overfed cows compared with controls (*p* < 0.001).

### 3.3. Calf Growth, Morphometric Traits, and Metabolic Responses

#### 3.3.1. Growth Performance

Calf growth performance was influenced primarily by parity, sex, and genotype rather than maternal overfeeding (Table 3). Male calves had greater birth weight and weaning weight than female calves (*p* < 0.01). Calves born to younger- and mature-parity dams showed greater weaning weight and average daily gain (ADG) to weaning compared with calves born to heifers (*p* < 0.05).

Meat-quality-oriented (MQ) genotype calves exhibited lower weaning weight than growth-oriented genotype calves (*p* < 0.05). Maternal overfeeding did not significantly affect calf birth weight, weaning weight, or ADG.

#### 3.3.2. Morphometric Traits

Several morphometric traits differed according to parity and calf sex (Table 3). Male calves showed greater cannon bone circumference at birth and greater withers height and chest depth at weaning than females (*p* < 0.05).

Calves born to younger-parity dams exhibited greater chest width, pelvic width, rump width, and body length compared with calves born to heifers (*p* < 0.05). MQ-genotype calves showed reduced pelvic width and rump length at weaning compared with growth-genotype calves (*p* < 0.05).

Increasing dam age was negatively associated with several width-related morphometric traits at birth.

#### 3.3.3. Metabolic Parameters

At birth, calves from younger-parity dams had higher albumin concentrations and lower glucose concentrations than calves from older dams (*p* < 0.05). Calves born to overfed dams exhibited lower NEFA concentrations at birth (*p* < 0.05).

Blood urea nitrogen (BUN) concentrations at birth and weaning were higher in calves from mature- and older-parity dams than in calves from heifers (*p* < 0.05). Male calves showed lower BUN and total protein concentrations at weaning than female calves (*p* < 0.05).

Most metabolic indicators at weaning were not significantly affected by maternal nutritional treatment.

### 3.4. Machine Learning Prediction Models

#### 3.4.1. Prediction of Maternal Outcomes

Machine learning models demonstrated moderate predictive performance for postpartum maternal traits (Table 4). For postpartum BCS, random forest showed the highest predictive accuracy (R^2^ = 0.557), followed by linear regression and XGBoost. For days to estrus return, random forest also showed the highest R^2^ value (0.449), whereas XGBoost produced the lowest RMSE.

**Figure 1:**
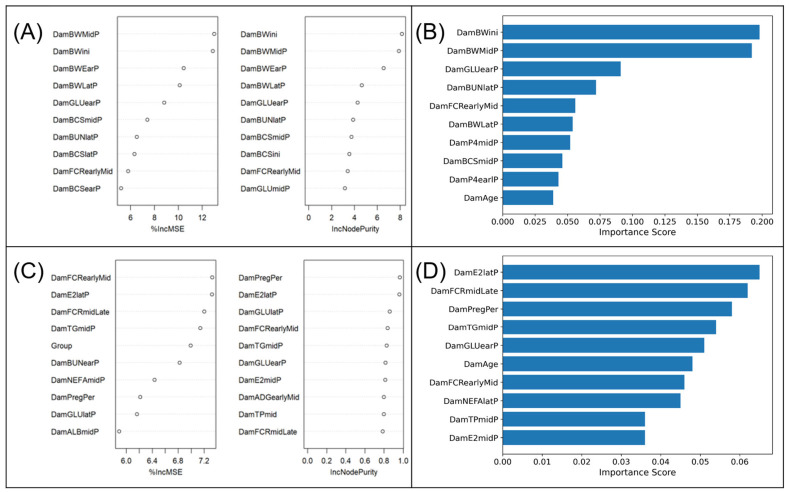
Feature importance rankings for postpartum body condition score (BCS) and days to estrus return in Hanwoo cows identified using machine learning models. (**A**,**C**) Random forest variable importance based on %IncMSE and IncNodePurity for postpartum BCS and estrus return interval, respectively. (**B**,**D**) XGBoost feature importance rankings based on gain values for postpartum BCS and estrus return interval, respectively. Gestational body weight, feed efficiency traits, and metabolic indicators were among the major predictors of postpartum outcomes. Variable abbreviations are described in Supplementary Table S3.

Feature importance analyses indicated that gestational BW measurements, feed efficiency traits, and metabolic indicators were among the most influential predictors of postpartum outcomes (Figure 1). Variables related to gestational body condition and lipid metabolism contributed substantially to model performance.

#### 3.4.2. Prediction of Calf Outcomes

For calf growth traits, XGBoost achieved the highest predictive performance among the evaluated models (Table 4). Prediction accuracy was highest for weaning weight (R^2^ = 0.978) and ADG to weaning (R^2^ = 0.921), whereas prediction of birth weight showed relatively low accuracy across models (R^2^ ≤ 0.274).

**Figure 2:**
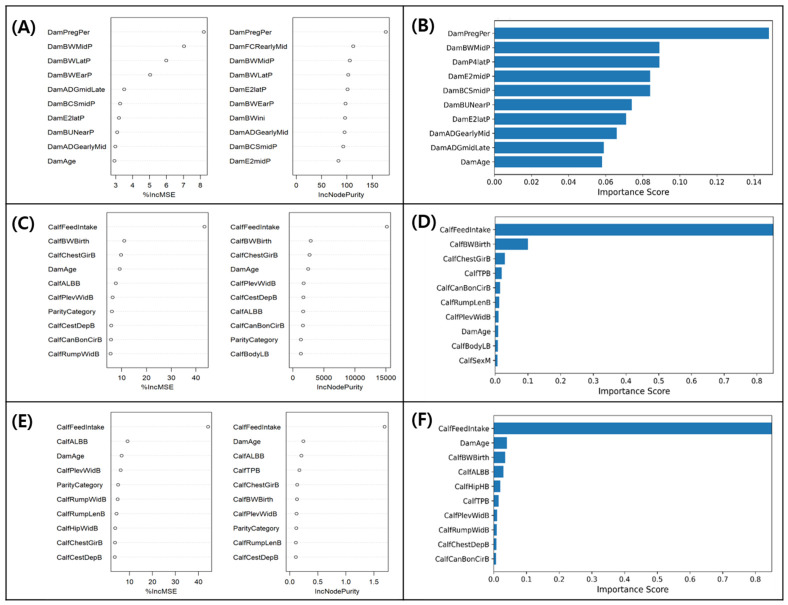
Feature importance rankings for calf birth weight (BW), weaning weight (WW), and average daily gain to weaning (ADGW) identified using machine learning models. (**A**,**C**,**E**) Random forest variable importance based on %IncMSE and IncNodePurity for BW, WW, and ADGW, respectively. (**B**,**D**,**F**) XGBoost feature importance rankings based on gain values for BW, WW, and ADGW, respectively. Maternal gestational traits, calf feed intake, morphometric measurements, and metabolic indicators were identified as major predictors of calf growth performance. Variable abbreviations are described in Supplementary Table S3.

Feature importance analyses identified calf feed intake, birth weight, morphometric traits, dam age, and metabolic indicators as major contributors to prediction accuracy for weaning performance (Figure 2). Maternal gestational BW and selected metabolic variables were among the major predictors of calf birth weight.

## 4. Discussion

The present study evaluated the effects of maternal overfeeding during mid-to-late gestation on maternal physiology, reproductive recovery, and calf performance in Hanwoo cattle using both conventional statistical analyses and machine learning (ML) approaches. Overall, maternal overfeeding improved gestational body condition and energy status, whereas calf growth responses were more strongly influenced by parity, sex, and genotype than by nutritional treatment alone.

Overfed cows showed increased body condition score (BCS) and average daily gain (ADG) during gestation, together with reduced circulating non-esterified fatty acid (NEFA) concentrations. Lower NEFA concentrations generally indicate reduced lipid mobilization and improved energy balance during late gestation [20]. These findings agree with previous studies reporting that strategic nutritional supplementation during gestation improves nutrient utilization and maternal metabolic stability in beef cattle [8,17]. Increased BCS in overfed cows further suggests enhanced energy reserve accumulation during pregnancy.

Interestingly, glucose concentrations during late gestation were lower in overfed cows despite the greater nutrient supply. Although high-energy feeding is commonly associated with increased circulating glucose concentrations, similar reductions have occasionally been reported under conditions of altered maternal insulin sensitivity and increased nutrient partitioning during late gestation [34]. The reduced glucose concentrations observed in the present study may therefore reflect altered maternal metabolic adaptation and potentially increased fetal glucose utilization during pregnancy. However, placental glucose transport activity and fetal metabolic measurements were not evaluated in the present study, and therefore this interpretation should be considered cautiously.

Parity also substantially influenced maternal performance. Younger-parity cows showed greater body weight gain and improved feed efficiency compared with older cows, indicating greater metabolic responsiveness to nutritional supply. Previous studies have similarly reported reduced adaptive capacity and nutrient utilization efficiency in older cows due to cumulative physiological demands associated with repeated reproduction [21,35]. These results suggest that parity should be considered when designing nutritional management strategies for pregnant beef cows.

Despite improved maternal body condition, overfed cows exhibited a longer interval to postpartum estrus return. Excessive energy intake and rapid body condition gain during late gestation may negatively influence endocrine regulation associated with reproductive recovery [20,36,37]. Previous studies have suggested that excessive nutritional intake during late gestation may alter hepatic steroid metabolism, insulin sensitivity, and luteinizing hormone pulsatility associated with postpartum ovarian cyclicity [34,36,37]. Although moderate improvements in BCS are generally associated with enhanced reproductive performance, excessive energy accumulation before calving may delay the resumption of ovarian cyclicity [20,36]. Therefore, the present findings support the concept that optimal rather than maximal nutrient supply may be more beneficial for postpartum reproductive efficiency.

In calves, maternal overfeeding did not significantly affect birth weight or overall growth performance to weaning. Instead, calf growth traits were more strongly associated with parity, sex, and genotype. Male calves and calves born to younger- and mature-parity dams showed greater weaning weight and average daily gain, consistent with previous studies describing sex- and parity-related differences in growth potential and maternal support capacity [21,38,39]. These findings suggest that biological factors may partially override the direct effects of maternal nutritional treatment on calf growth performance. In addition, excessive nutritional supply during gestation may primarily influence maternal metabolic adaptation rather than directly enhancing offspring growth performance. Therefore, the present findings indicate that increased maternal nutrient intake alone may not necessarily result in proportional improvements in calf growth under all production conditions.

Although overfeeding did not significantly increase calf growth, calves born to overfed dams showed lower NEFA concentrations at birth, suggesting improved neonatal energy status. However, these metabolic differences were not maintained until weaning, indicating that postnatal nutrition and management likely exert a greater influence on long-term calf growth than prenatal nutritional treatment alone [40,41]. Similar inconsistencies between prenatal nutritional intervention and postnatal performance have been reported previously [17,18,19].

Machine learning analyses further demonstrated that gestational body weight trajectories, metabolic indicators, feed efficiency, and calf genotype were important predictors of maternal and calf outcomes. Random forest showed superior predictive performance for maternal traits, whereas XGBoost performed best for calf growth traits. These findings are consistent with previous studies demonstrating that ensemble ML approaches effectively model complex biological interactions in livestock datasets [24,25,26,27,28]. Although conventional regression models may perform adequately when relationships among variables are predominantly linear, machine learning approaches may provide additional advantages for capturing nonlinear interactions among nutritional, physiological, and genetic factors.

Prediction accuracy for calf birth weight was relatively low compared with weaning traits, suggesting that additional variables related to placental function, early fetal development, or genomic background may be required to better explain neonatal growth variation. In contrast, weaning weight and ADG showed high predictive accuracy, likely reflecting the contribution of biologically relevant postnatal variables, including feed intake and morphometric development measured during the pre-weaning period. Nevertheless, the possibility of partial overfitting cannot be completely excluded. 

Several limitations should be considered when interpreting the present findings. First, the study was conducted exclusively in Hanwoo cattle under a specific production system, which may limit direct extrapolation to other beef breeds or management conditions. In addition, external validation using independent populations was not performed, and therefore further studies are required to confirm the generalizability of the developed prediction models. Furthermore, the physiological and productive characteristics of Hanwoo cattle may differ from those of other commercial beef breeds, which could influence the transferability of the developed prediction models across production systems. In addition, the absence of a pair-fed control group limited the ability to completely separate the effects of increased feed intake from nutrient density or energy supply.

Collectively, the present study demonstrates that maternal overfeeding during gestation primarily influences maternal metabolic adaptation rather than directly enhancing calf growth performance. Furthermore, parity, calf sex, and genotype emerged as major biological factors associated with productivity-related traits. Integration of nutritional management with ML-based prediction models may therefore contribute to precision feeding and reproductive management strategies in beef cattle production systems.

## 5. Conclusions

This study demonstrated that maternal overfeeding during mid-to-late gestation altered maternal metabolic adaptation and reproductive responses in Hanwoo cattle. Overfeeding increased body condition score (BCS) and gestational average daily gain (ADG) while reducing circulating non-esterified fatty acid (NEFA) concentrations, indicating improved maternal energy status during late gestation. However, overfed cows showed a longer interval to postpartum estrus return, suggesting that excessive nutrient supply during gestation may not necessarily improve reproductive recovery.

In calves, maternal overfeeding had limited effects on birth weight and overall growth performance to weaning. Instead, parity, calf sex, and genotype were more strongly associated with calf growth, morphometric development, and metabolic traits. These findings indicate that biological factors may exert a greater influence on calf productivity than maternal nutritional treatment alone.

Machine learning analyses further identified gestational body weight trajectories, metabolic indicators, feed efficiency, and calf genotype as important predictors of maternal and calf outcomes. Random forest and XGBoost models showed strong predictive performance for several productivity-related traits, supporting the potential application of data-driven approaches in beef cattle management.

Collectively, these results suggest that parity- and genotype-informed nutritional management, combined with machine learning-based prediction models, may serve as complementary tools supporting precision feeding and reproductive management strategies in beef cattle production systems. However, the absence of a pair-fed control group limited the ability to completely separate the effects of increased feed intake from nutrient density or energy supply. In addition, the study was conducted exclusively in Hanwoo cattle under a specific production system, which may limit direct extrapolation to other beef breeds or management conditions. Future studies using independent external datasets will be necessary to validate the robustness and practical applicability of the developed machine learning models under diverse production environments.

## Figures and Tables

**Table 1:** Nutrient composition of concentrate and rice straw used in the experimental diets.

Nutrient	Concentrate	Rice Straw
Dry matter (DM) (%)	92.8	82.3
Crude protein (% DM)	16.7	5.0
Total digestible nutrients (% DM)	63.2	29.8
Neutral detergent fiber (% DM)	31.8	62.1
Acid detergent fiber (% DM)	17.4	41.0
Ash (% DM)	10.0	13.5
Ether extract (% DM)	4.2	1.7
Crude fiber (% DM)	12.4	37.9
Lignin (% DM)	6.4	6.5

Abbreviations: DM, dry matter; TDN, total digestible nutrients; NDF, neutral detergent fiber; ADF, acid detergent fiber.

**Table 2:** Effects of parity, nutritional treatment, and dam age on maternal growth, metabolic, and reproductive traits in Hanwoo cows.

Outcome	Parity Effect (*p*-Value)	Overfeeding (GroupT) Effect (*p*-Value)	Dam Age Effect (*p*-Value)
Body weight and growth performance			
BWMidP	Young (↑ 0.0047)	—	—
BWLatP	Young (↑ 0.023)	↑ 0.0001	—
BWAftP	Young (↑ 0.018)	—	↑ 0.025
ADGmidLate	Young (↑ 0.026)	↑ 0.001	↓ 0.00051
FCRmidLate	Young (↓ 0.0022)	↓ 0.0094	↑ 0.016
Body condition score (BCS)			
BCSmidP	—	↑ 0.00028	—
BCSlatP	—	↑ 0.001	—
Metabolic indicators			
ALBmidP	Young (↑ 0.028)	↓ 0.027	↓ 0.00019
ALBlatP	—	—	↑ 0.001
TGmidP	—	—	↑ 0.011
TGlatP	—	↑ 0.0068	↑ 0.026
BUNmidP	—	—	↑ 0.001
TPmidP	—	↓ 0.001	↑ 0.001
CHOmidP	—	↑ 0.014	↑ 0.001
CHOlatP	Young (↓ 0.049), Mature (↓ 0.039)	↑ 0.0021	↑ 0.018
GLUlatP	—	↓0.0002	↓ 0.0096
NEFAmidP	Mature (↓ 0.030)	↓ 0.017	↑ 0.001
NEFAlatP	Young (↓ 0.000028), Mature (↓ 0.023)	↓ 0.0095	—
Reproductive parameters			
EstRet	—	↑ 0.00073	—
P4midP	—	↓ 0.00034	—

Significant increasing and decreasing effects are indicated by ↑ and ↓, respectively (*p* < 0.05). Parity effects are presented relative to the heifer group as the reference category. BWMidP, body weight during mid gestation; BWLatP, body weight during late gestation; BWAftP, body weight postpartum; ADGmidLate, average daily gain from mid-to-late gestation; FCRmidLate, feed conversion ratio from mid-to-late gestation; BCS, body condition score; ALB, albumin; TG, triglycerides; BUN, blood urea nitrogen; TP, total protein; CHO, cholesterol; GLU, glucose; NEFA, non-esterified fatty acids; EstRet, postpartum estrus return interval; P4midP, progesterone concentration during mid gestation. MidP, mid gestation; LatP, late gestation; AftP, postpartum. Sample sizes varied among analyses because missing observations occurred due to unsuccessful blood collection, incomplete laboratory measurements, or exclusion of animals with incomplete experimental records.

**Table 3:** Effects of parity, maternal overfeeding, calf sex, genotype, and dam age on calf growth, morphometric, and metabolic traits.

Outcome	Parity Effect (*p*-Value)	Overfeeding Effect (*p*-Value)	Sex Effect (*p*-Value)	Genotype Effect (MQ, *p*-Value)	Dam Age Effect (*p*-Value)
Growth performance					
BWBirth	—	—	Male (↑ 0.001)	—	—
CalfBWW	Young (↑ 0.001), Mature (↑ 0.011)	—	Male (↑ 0.001)	MQ (↓ 0.025)	—
CalfADGW	Young (↑ 0.003), Mature (↑ 0.010)	—	Male (↑ 0.009)	—	—
FeedIntake	Young (↓ 0.001), Mature (↓ 0.013)	—	Male (↓ 0.012)	—	—
Morphometric measurements					
CanBonCirB	—	—	Male (↑ 0.001)	—	—
WitHW	—	—	Male (↑ 0.007)	—	—
BodLW	Young (↑ 0.002), Mature (↑ 0.041)	—	—	—	—
ChestGirB	—	↓ 0.033	Male (↑ 0.011)	—	—
ChestWidB	Young (↑ 0.001)	—	—	—	↓ 0.001
ChestGirW	Young (↑ 0.020)	—	Male (↑ 0.023)	—	—
ChestDepW	Young (↑ 0.001), Mature (↑ 0.032)	—	Male (↑ 0.022)	—	—
PlevWidB	Young (↑ 0.003)	—	—	—	↓ 0.022
PlevWidW	Young (↑ 0.001), Mature (↑ 0.023)	—	—	MQ (↓ 0.050)	—
HipWidB	—	—	—	—	↓ 0.029
HipWidW	Young (↑ 0.001)	—	—	—	—
RumpWidB	Young (↑ 0.01)	—	—	—	↓ 0.021
RumpWidW	Young (↑ 0.001)	—	—	—	—
RumpLenW	Young (↑ 0.001), Mature (↑ 0.035)	—	—	MQ (↓ 0.014)	—
Metabolic indicators					
ALBB	Young (↑ 0.001)	—	—	—	↓ 0.005
ALBW	Young (↑ 0.019)	—	—	—	—
GLUB	Young (↓ 0.044)	—	Male (↓ 0.020)	—	—
CHOB	Young (↑ 0.009)	—	—	—	—
BUNB	Young (↑ 0.031), Mature (↑ 0.002), Older (↑ 0.009)	—	—	—	↓ 0.015
BUNW	Young (↑ 0.010), Mature (↑ 0.008), Older (↑ 0.019)	—	Male (↓ 0.001)	—	↓ 0.040
TPB	—	—	—	—	↑ 0.022
TPW	—	—	Male (↓ 0.009)	—	—
NEFAB	—	↓ 0.037	—	—	—
TGW	Mature (↓ 0.045)	—	—	—	—

Significant increasing and decreasing effects are indicated by ↑ and ↓, respectively (*p* < 0.05). Parity effects are presented relative to the heifer group as the reference category. MQ indicates meat-quality-oriented genotype, whereas the reference category represents growth-oriented genotype calves.

**Table 4:** Predictive performance of machine learning models for maternal and calf productivity traits.

Trait	Model	R^2^	RMSE
Postpartum BCS in cows	LM	0.502	0.438
	RF	0.557	0.421
	XGB	0.475	0.430
Days to estrus return in cows	LM	0.398	0.335
	RF	0.449	0.324
	XGB	0.425	0.320
ADG to weaning in calves	LM	0.905	0.0358
	RF	0.853	0.0382
	XGB	0.921	0.0326
Calves birth weight	LM	0.267	3.504
	RF	0.241	3.627
	XGB	0.274	3.485
Calves weaning weight	LM	0.964	1.920
	RF	0.908	2.458
	XGB	0.978	1.776

## Data Availability

The data presented in this study are available on reasonable request from the corresponding authors. The data are not publicly available due to institutional and ethical restrictions.

## Supplementary Materials

The following supporting information can be downloaded at: https://www.mdpi.com/article/10.3390/ani16121902/s1. Table S1: Descriptive statistics for maternal growth, metabolic, and reproductive traits; Table S2: Descriptive statistics for calf growth, morphometric, and metabolic traits; Table S3: Abbreviation list for maternal and calf variables; Table S4: Summary of significant predictors associated with maternal outcomes identified by robust regression models; Table S5: Definitions of calf growth, morphometric, nutritional, and metabolic variables used in the analyses.
