# Supplementary material for: Impact of Mid-to-Late Gestational Overfeeding on Maternal Performance and Calf Outcomes in Hanwoo Cattle: A Machine Learning Approach
Source: Animals (Basel). 2026 Jun 19;16(12):1902. doi: 10.3390/ani16121902 (PMC13295325; doi:10.3390/ani16121902)
Supplement: Supplementary file 1 [file animals-16-01902-s001.zip › animals-4360649-supplementary.pdf]

Table S1. Descriptive statistics for maternal growth, metabolic, and reproductive traits

Variable	Mean	SD	Min	Q1	Median	Q3	Max
DamADGearlyMid	0.43	0.25	0.00	0.23	0.40	0.58	1.48
DamADGmidLate	0.58	0.32	0.00	0.35	0.58	0.71	1.61
DamALBearP	3.14	0.24	2.30	3.00	3.10	3.30	3.90
DamALBlatP	3.18	0.33	0.10	3.10	3.20	3.30	4.00
DamALBmidP	3.13	0.27	2.10	3.00	3.10	3.30	3.90
DamBCSAfP	3.19	0.61	1.70	3.00	3.00	3.50	5.00
DamBCSearP	3.14	0.54	1.50	2.83	3.10	3.50	4.50
DamBCSini	3.08	0.51	1.50	2.75	3.00	3.30	4.40
DamBCSlatP	3.27	0.64	1.00	3.00	3.50	3.50	5.00
DamBCSmidP	3.31	0.53	1.90	3.00	3.31	3.67	4.50
DamBUNearP	14.90	5.27	4.60	10.43	15.25	18.58	51.50
DamBUNlatP	13.13	3.06	5.20	10.80	12.95	15.17	22.90
DamBUNmidP	15.06	3.71	8.10	13.03	14.85	16.80	51.60
DamBWAftP	453.25	74.52	237.00	407.50	453.50	497.75	699.00
DamBWEarP	401.26	72.43	216.00	350.25	395.00	445.00	676.00
DamBWLatP	472.75	77.65	283.00	420.00	475.00	523.50	746.00
DamBWMidP	438.35	69.87	265.00	389.25	436.50	487.75	706.00
DamBWini	382.36	56.79	249.00	356.50	383.27	403.00	542.00
DamCHOearP	106.87	22.93	33.00	91.00	106.00	120.00	197.00
DamCHOlatP	117.54	19.44	66.00	105.00	114.00	131.00	205.00
DamCHOmidP	120.42	23.02	56.00	106.00	119.00	135.75	219.00
DamE2earP	76.20	87.17	0.00	38.09	54.65	84.84	913.81

DamE2latP	107.21	82.14	6.66	40.46	87.59	162.98	416.47
DamE2midP	114.45	67.73	8.31	60.00	103.81	146.38	324.18
DamEstRet	42.23	15.78	11.00	28.00	43.00	50.75	87.00
DamFCRearlyMid	35.82	46.04	5.01	15.57	21.38	33.83	456.75
DamFCRmidLate	30.76	49.87	5.73	12.69	19.85	30.58	555.75
DamGLUearP	44.08	14.42	8.00	34.00	47.50	55.00	73.00
DamGLUlatP	39.97	12.34	9.00	30.00	40.00	48.00	109.00
DamGLUmidP	37.44	10.40	9.00	30.00	38.00	44.00	72.00
DamNEFAearP	139.46	124.13	20.00	70.00	95.00	150.00	740.00
DamNEFAlatP	254.46	176.79	30.00	130.00	210.00	330.00	1,080.00
DamNEFAmidP	127.02	90.59	10.00	70.00	90.00	150.00	570.00
DamP4earlP	49.24	57.59	0.02	19.12	39.78	49.06	594.44
DamP4latP	42.32	24.29	0.05	24.98	39.69	51.86	143.37
DamP4midP	35.08	30.75	0.89	13.60	25.48	43.79	162.68
DamPregPer	285.27	9.02	258.00	280.00	284.00	288.75	325.00
DamTGearP	15.25	5.09	5.00	12.00	15.00	18.00	31.00
DamTGlatP	18.31	7.63	4.00	13.00	17.00	22.00	49.00
DamTGmidP	15.33	8.02	1.00	10.00	14.00	19.00	64.00
DamTPearP	7.30	0.49	5.80	7.00	7.20	7.60	8.80
DamTPlatP	7.44	0.49	5.70	7.10	7.40	7.80	8.80
DamTPmid	7.66	0.50	6.60	7.30	7.60	7.90	9.30

Table S2. Descriptive statistics for calf growth, morphometric, and metabolic traits

Variable	Mean	SD	Min	Q1	Median	Q3	Max
CalfADGW	0.60	0.13	0.18	0.52	0.61	0.68	1.00

CalfALBB	2.37	0.64	1.50	1.90	2.10	3.10	3.60
CalfALBW	3.08	0.22	2.40	3.00	3.10	3.20	3.70
CalfBUNB	9.96	4.51	2.70	7.23	9.40	11.80	44.50
CalfBUNW	15.33	4.41	6.60	12.20	15.05	18.17	43.80
CalfBWBirth	26.37	4.28	17.00	23.25	26.00	29.00	45.00
CalfBWW	82.25	13.96	45.00	74.00	83.00	90.50	139.00
CalfBodLW	83.86	6.71	63.00	79.25	85.00	88.00	102.00
CalfBodyLB	56.71	4.79	22.00	54.00	57.00	59.00	71.00
CalfCHOB	81.14	51.54	17.00	42.00	61.50	121.75	287.00
CalfCHOW	136.93	28.54	76.00	118.00	134.50	155.00	244.00
CalfCanBonCirB	9.98	0.69	8.50	9.50	10.00	10.38	12.00
CalfCesDepW	38.21	2.85	32.00	36.00	38.00	40.00	47.00
CalfCestDepB	25.62	2.94	11.00	24.00	25.00	27.00	39.00
CalfChestGirB	67.76	4.15	55.00	66.00	68.00	70.00	82.00
CalfChestGirW	98.95	8.81	26.00	96.00	100.00	103.00	118.00
CalfChestWidB	13.26	2.11	10.00	12.00	13.00	15.00	20.00
CalfChestWidW	18.72	2.65	11.00	17.00	19.00	20.38	25.00
CalfFeedIntake	1.79	0.55	0.99	1.46	1.65	1.95	5.50
CalfGLUB	89.70	33.35	3.00	65.00	95.50	111.00	186.00
CalfGLUW	89.13	20.92	42.00	76.00	90.00	100.75	207.00
CalfHipHB	70.20	4.53	47.00	67.00	70.00	73.00	87.00
CalfHipHW	88.13	4.34	74.00	85.00	88.25	91.00	104.00
CalfHipWidB	8.81	2.00	6.00	7.00	8.00	10.00	18.00
CalfHipWidW	14.82	2.23	9.00	13.00	15.00	16.38	22.00
CalfNEFAB	263.66	141.51	0.34	180.00	230.00	300.00	1,060.00

CalfNEFAW	233.26	137.54	70.00	150.00	200.00	260.00	1,009.00
CalfPlevWidB	16.20	2.28	12.00	15.00	16.00	18.00	24.00
CalfPlevWidW	23.03	2.81	14.00	21.00	23.00	25.00	30.00
CalfRumpLenB	19.83	2.20	8.00	18.00	20.00	21.00	30.00
CalfRumpLenW	28.41	3.11	15.00	26.00	28.75	30.50	37.00
CalfRumpWidB	13.03	2.16	9.00	11.00	12.25	15.00	19.00
CalfRumpWidW	19.35	3.05	12.00	17.50	19.50	21.50	36.00
CalfTGB	42.98	28.73	1.00	21.25	37.50	58.75	241.00
CalfTGW	18.75	10.41	5.00	11.00	16.00	24.00	76.00
CalfTPB	6.74	3.15	3.80	5.80	6.40	7.38	52.00
CalfTPW	6.38	0.45	5.40	6.10	6.30	6.60	8.60
CalfWitHB	67.12	4.04	57.00	64.00	67.00	70.00	84.00
CalfWitHW	85.06	3.93	70.00	82.00	85.00	88.00	97.00

Table S3. Abbreviation list for maternal and calf variables

Abbreviation	Full Definition
DamADGearlyMid	Maternal average daily gain from early to mid gestation
DamADGmidLate	Maternal average daily gain from mid to late gestation
DamALBearP	Maternal serum albumin concentration during early gestation
DamALBmidP	Maternal serum albumin concentration during mid gestation
DamALBlatP	Maternal serum albumin concentration during late gestation
DamBCSini	Initial maternal body condition score
DamBCSearP	Maternal body condition score during early gestation
DamBCSmidP	Maternal body condition score during mid gestation
DamBCSlatP	Maternal body condition score during late gestation
DamBCSAfP	Maternal body condition score after parturition
DamBUNearP	Maternal blood urea nitrogen concentration during early gestation
DamBUNmidP	Maternal blood urea nitrogen concentration during mid gestation
DamBUNlatP	Maternal blood urea nitrogen concentration during late gestation
DamBWini	Initial maternal body weight
DamBWEarP	Maternal body weight during early gestation
DamBWMidP	Maternal body weight during mid gestation
DamBWLatP	Maternal body weight during late gestation
DamBWAftP	Maternal body weight after parturition

DamCHOearP	Maternal serum cholesterol concentration during early gestation
DamCHOmidP	Maternal serum cholesterol concentration during mid gestation
DamCHOlatP	Maternal serum cholesterol concentration during late gestation
DamE2earP	Maternal estradiol concentration during early gestation
DamE2midP	Maternal estradiol concentration during mid gestation
DamE2latP	Maternal estradiol concentration during late gestation
DamEstRet	Days to postpartum estrus return
DamFCRearlyMid	Maternal feed conversion ratio from early to mid gestation
DamFCRmidLate	Maternal feed conversion ratio from mid to late gestation
DamGLUearP	Maternal serum glucose concentration during early gestation
DamGLUmidP	Maternal serum glucose concentration during mid gestation
DamGLUlatP	Maternal serum glucose concentration during late gestation
DamNEFAearP	Maternal serum non-esterified fatty acid concentration during early gestation
DamNEFAmidP	Maternal serum non-esterified fatty acid concentration during mid gestation
DamNEFAlatP	Maternal serum non-esterified fatty acid concentration during late gestation
DamP4earP	Maternal progesterone concentration during early gestation
DamP4midP	Maternal progesterone concentration during mid gestation
DamP4latP	Maternal progesterone concentration during late gestation
DamPregPer	Pregnancy period
DamTGearP	Maternal serum triglyceride concentration during early gestation
DamTGmidP	Maternal serum triglyceride concentration during mid gestation
DamTGlatP	Maternal serum triglyceride concentration during late gestation
DamTPearP	Maternal total protein concentration during early gestation
DamTPmid	Maternal total protein concentration during mid gestation
DamTPlatP	Maternal total protein concentration during late gestation
CalfADGW	Calf average daily gain to weaning
CalfALBB	Calf serum albumin concentration at birth
CalfALBW	Calf serum albumin concentration at weaning
CalfBUNB	Calf blood urea nitrogen concentration at birth
CalfBUNW	Calf blood urea nitrogen concentration at weaning
CalfBWBirth	Calf body weight at birth
CalfBWW	Calf body weight at weaning
CalfBodyLB	Calf body length at birth
CalfBodLW	Calf body length at weaning
CalfCHOB	Calf serum cholesterol concentration at birth
CalfCHOW	Calf serum cholesterol concentration at weaning
CalfCanBonCirB	Calf cannon bone circumference at birth
CalfCestDepB	Calf chest depth at birth
CalfCesDepW	Calf chest depth at weaning
CalfChestGirB	Calf chest girth at birth
CalfChestGirW	Calf chest girth at weaning
CalfChestWidB	Calf chest width at birth
CalfChestWidW	Calf chest width at weaning
CalfFeedIntake	Calf feed intake during preweaning period
CalfGLUB	Calf serum glucose concentration at birth

CalfGLUW	Calf serum glucose concentration at weaning
CalfHipHB	Calf hip height at birth
CalfHipHW	Calf hip height at weaning
CalfHipWidB	Calf hip width at birth
CalfHipWidW	Calf hip width at weaning
CalfNEFAB	Calf serum non-esterified fatty acid concentration at birth
CalfNEFAW	Calf serum non-esterified fatty acid concentration at weaning
CalfPlevWidB	Calf pelvic width at birth
CalfPlevWidW	Calf pelvic width at weaning
CalfRumpLenB	Calf rump length at birth
CalfRumpLenW	Calf rump length at weaning
CalfRumpWidB	Calf rump width at birth
CalfRumpWidW	Calf rump width at weaning
CalfTGB	Calf serum triglyceride concentration at birth
CalfTGW	Calf serum triglyceride concentration at weaning
CalfTPB	Calf total protein concentration at birth
CalfTPW	Calf total protein concentration at weaning
CalfWitHB	Calf withers height at birth
CalfWitHW	Calf withers height at weaning

Table S4. Summary of significant predictors associated with maternal outcomes identified by robust regression models

Outcome	Significant predictor	Effect direction	Estimate	p-value
Maternal ADG (mid–late gestation)	Overfeeding	↑	0.111	<0.001
Maternal BCS (late gestation)	Overfeeding	↑	0.104	<0.001
Maternal glucose (late gestation)	Overfeeding	↓	-0.136	<0.001
Maternal NEFA (late gestation)	Overfeeding	↓	-0.224	0.009
Postpartum estrus return interval	Overfeeding	↑	—	<0.01

Table S5. Definitions of calf growth, morphometric, nutritional, and metabolic variables used in the analyses

Variable	Trait Category	Timepoint	Definition
CalfBWBirth	Growth trait	Birth	Body weight at birth (kg)

CalfBWW	Growth trait	Weaning	Body weight at weaning (kg)
CalfADGW	Growth trait	Birth–weaning	Average daily gain to weaning (kg/day)
CalfFeedIntake	Nutritional trait	Preweaning	Feed intake during preweaning period (kg/day)
CalfChestWidB	Morphometric trait	Birth	Chest width at birth (cm)
CalfChestWidW	Morphometric trait	Weaning	Chest width at weaning (cm)
CalfHipHW	Morphometric trait	Weaning	Hip height at weaning (cm)
CalfRumpLenW	Morphometric trait	Weaning	Rump length at weaning (cm)
CalfGLUB	Metabolic trait	Birth	Serum glucose concentration at birth (mg/dL)
CalfGLUW	Metabolic trait	Weaning	Serum glucose concentration at weaning (mg/dL)
CalfNEFAB	Metabolic trait	Birth	Serum non-esterified fatty acid concentration at birth (μEq/L)
CalfNEFAW	Metabolic trait	Weaning	Serum non-esterified fatty acid concentration at weaning (μEq/L)
